# Risky behavior in virtual reality: The roles of personality, environment, and physiology

**DOI:** 10.1371/journal.pone.0316896

**Published:** 2025-01-13

**Authors:** Dejan Pajić, Selka Sadiković, Milan Oljača, Željko Popović, Lazar Milić, Goran Stojanović, Snežana Smederevac

**Affiliations:** 1 Faculty of Philosophy, Department of Psychology, University of Novi Sad, Novi Sad, Serbia; 2 Faculty of Technical Sciences, Department of Electronics, University of Novi Sad, Novi Sad, Serbia; University of Bucharest, Faculty of Biology, ROMANIA

## Abstract

Virtual reality (VR) provides a unique opportunity to simulate various environments, enabling the observation of human behavior in a manner that closely resembles real-world scenarios. This study aimed to explore the effects of anticipating reward or punishment, personality traits, and physiological arousal on risky decision-making within a VR context. A custom VR game was developed to simulate real-life experiences. The sample comprised 52 students (63.46% female) from the University of Novi Sad, Serbia. The study assessed four parameters within the VR environment: elapsed game time, number of steps taken, average score, and decision-making time. Three physiological signals, heart rate, skin conductance, and respiratory rate, were recorded. Results indicated that personality traits, specifically Fight (β = -0.33, p = 0.024) and Freeze (β = 0.431, p = 0.009), were significantly related to behavior in the VR environment (R = 0.572, R^2^_adj = 0.227, RMSE = 23.12, F(6, 40) = 3.25, p = 0.011). However, these effects were not significant after negative feedback. Emotional arousal, measured by respiratory rate amplitude (β = 0.276, p = 0.045), showed a more pronounced role after feedback (β = 0.337, p = 0.028). These findings indicate that personality traits primarily influence behavior in a VR environment prior to the actual threat, whereas environmental characteristics become more important afterwards. The results offer valuable insights for experimental and personality psychologists by revealing how risk-taking is influenced by situational, emotional, and personality factors. Additionally, they provide guidance for VR designers in creating more ecologically valid environments, highlighting VR’s potential as a tool for psychological research, while also underscoring the critical importance of selecting objective VR measures to accurately capture the complexities of human behavior in immersive environments.

## Introduction

Risky decision-making refers to the process of selecting choices within uncertain scenarios where both positive and negative outcomes are plausible. In such circumstances, individuals must assess the trade-offs between potential rewards and risks before making a choice [1]. The decision-making framework is usually described by three types of framing tasks: risky choice framing, attribute framing, and goal framing [2]. Framing manipulation determines whether expected outcomes are evaluated in terms of gains or losses, with most subjects exhibiting risk aversion in the positive framing condition and risk-seeking behavior in negative framing condition [3]. In essence, individuals are more inclined to take risks when there is a higher likelihood of avoiding losses than making gains, as demonstrated in the *Asian Disease Problem* [3].

Attribute framing can lead to different decisions based on the valence of presented information. People tend to be more risk-averse when information is positively framed and more risk-seeking when presented in a negative frame [2]. Goal framing involves decision-making influenced by the description of goals, where positive framing directs attention to the goal of obtaining a positive consequence, while negative framing directs attention to avoiding a negative consequence [2]. Beyond decision features, risky decision-making is shaped by numerus factors that can be considered as either individual or contextual [4].

### Individual differences: Revised Reinforcement Sensitivity Theory (rRST)

Previous research has demonstrated individual differences in tendency to take risks and make risky decisions [5]. Examples of impulsive activities, traditionally associated with risk-taking tendencies, range from socially unacceptable behaviors like drug use, drunk driving, and unsafe sexual practices, to more socially acceptable pursuits such as extreme sports or adventure travelling. Therefore, it is plausible to assume that prior experiences and stable personality traits play a significant role in shaping responses to specific situations. The prevailing trend in previous studies suggests that individuals with a heightened inclination towards impulsivity or sensation-seeking tend to opt for riskier decisions more frequently [6]. However, it is crucial to recognize that various personality traits can shape the manifestation of tendency toward risky behavior and their examination is paramount for understanding this phenomenon. For instance, emotions play an important role in decision-making, since decisions can serve as a means to avoid negative feelings like guilt and regret, while enhancing positive emotions such as pride and happiness [7]. Decisions involving risk have been associated with emotions such as anger and anxiety [8].

A personality model that provides a comprehensive framework for examining the decision-making process is the *Revised Reinforcement Sensitivity Theory* (rRST) [9]. According to the rRST, three systems reflect typical responses of organisms to environmental stimuli. *Anxiety*, regulated by the *Behavioral Inhibition System* (BIS), manifests as a reaction to potential threats, such as conflict of goals or perceived lack of resources to address possible dangers. *Impulsivity*, regulated by the *Behavioral Approach System* (BAS), represents a reaction to potential rewards, such as desire for immediate gratification or inability to prolong gratification for long-term goals. *Fear*, regulated by the *Fight/Flight/Freeze System* (FFFS), is a reaction to real, immediate, and intense threat, prompting specific defensive behaviors. FFFS reactions are contingent on the perceived defensive distance. For instance, when a threat is sufficiently distant, flight may be a viable choice. In contrast, when the threat is imminent and proximate, the reaction might be to freeze as a form of cognitive inactivation. The decision to fight occurs when there is an assessment that adequate psychological resources are available to contend with a threat.

The suitability of the rRST for the investigation of risky decision-making is evident in its ability to capture the interplay between environmental characteristics and learned behavioral patterns, contributing to the shaping of behavior in specific situations. By encompassing both environmental factors and individual differences, this model provides a more comprehensive understanding of how individuals respond within a specific context, as demonstrated by previous studies. For instance, using the *Game of Dice Task* (GDT) [10], findings indicate that individuals with high BAS (linked to impulsivity), and low BIS (linked to anxiety), are inclined to make riskier decisions following a positive outcome. In contrast, those with low BAS and high BIS tend to opt for risk-free decisions after experiencing a loss. These results suggest an interaction between personality traits and decision-making strategies, revealing the differential influence of rewards and punishments on risk-taking behavior [11].

In recent years, fMRI studies have yielded both behavioral and neuroimaging evidence establishing a connection of BIS/BAS scores and mental activation during decision-making processes, particularly among those with limited explorative decision-making skills. For example, individuals with low performance in explorative decision-making tend to exhibit higher BAS scores and engage in more explorative attempts [12]. These individuals demonstrate a tendency to rely less on systematic searching when faced with uncertain situations. Additionally, the BIS/BAS scores of this low-performance group show significant associations with their neural activations during explorative decision-making. Specifically, higher BAS scores are negatively linked to neural networks associated with reward-seeking, whereas higher BIS scores are negatively linked to neural networks responding to risk choice.

Previous studies have also shown that the inclination toward risk-taking decisions, specifically those aimed at achieving rewards, tends to decrease over the course of an individual’s life. In contrast, the propensity for risk taking decisions directed at avoiding punishment remains relatively stable across years. This divergence implies a crucial distinction in the dynamics of risky decision-making between the pursuit of gains and the avoidance of losses [1]. Consequently, situations that encompass both components—the potential for gains and the risk of losses—present a formidable challenge for decision-makers. The juxtaposition of these divergent patterns adds complexity to the decision-making process, requiring individuals to navigate the delicate balance between seeking rewards and averting negative consequences.

### Contextual factors: Virtual reality (VR) environment

Various situational factors and aspects of decision-making play pivotal roles in determining the level of risk individuals are willing to take. Factors like time pressure and social context significantly contribute to shaping the risk-taking behavior in a given situation. When studying risky decision-making in experimental settings, researchers must carefully consider the impact of contextual elements on specific decisions, including factors like attractiveness or gain value [13]. Furthermore, findings of previous research indicate that time pressure exerts differential effects on risk taking depending on the probability of losing [14]. Specifically, time pressure tends to increase the frequency of risk-taking when the likelihood of losing is high but decreases it when the probability of losing is low. Moreover, the scope of risk taking extends across various domains such as health, recreation, finance, ethics, and social interactions [15]. Recognizing these diverse domains underscores the importance of establishing appropriate contexts when investigating behaviors associated with decision-making and risk-taking.

Virtual reality (VR) offers a unique opportunity to simulate various environments, enabling the observation of reactions in a manner closely resembling real-world scenarios [16]. Notably, the neural mechanisms experienced by individuals immersed in VR exhibit similarities to those in real-life situations [17] which makes VR a valuable tool for studying human behavior. Beyond simulating realistic settings, VR enables comprehensive recording of user behavior during interactions with the virtual environment [18]. This makes VR particularly well-suited for measuring reaction time (RT), as individuals can engage with authentic risks in a controlled virtual space [19]. Importantly, VR enables the real-time assessment of both timely reactions and physiological responses [20]. The integration of these features positions VR as a powerful tool for studying human behavior, offering researchers an interactive and controlled platform to investigate reactions, response times, and physiological changes in immersive and realistic contexts.

Typically, risk preferences in VR environments are examined by administering implicit or explicit gambling tasks, in which participants can choose between relatively risky or safe options. For example, the *Spheres & Shield Maze Task* (SSMT) was used to examine risk- taking propensity, significantly associated with risk-related traits [21]. Using the *Balloon Analogue Risk Task* (BART), researchers have demonstrated that the overall score is significantly correlated with sensation-seeking [22]. The *Go/No-Go Simulator Driving Task* (G/NG-SDT) was employed to assess drivers’ risky decisions and related behavioral assessments at a situation-specific level. Results indicated that higher frequency of "Go" decisions were associated with an increased tendency for risk on the road and distinguishable risky behavioral patterns [23].

### Physiological sensitivity: Measures of arousal in experimental conditions

There are hypotheses, such as the *Somatic Marker Hypothesis* [24], suggesting that unconscious bodily states guide decision-making. These "somatic markers" involve physiological responses like heart rate or skin conductance responses, shaped by early experiences of pleasure or aversion. Over time, neural pathways associated with these markers can be activated by similar situations (secondary inducers), facilitating automatic decision-making. This is particularly relevant in complex scenarios where somatic markers aid decisions and counter excessive rational reasoning. The ventromedial prefrontal cortex is believed to play a crucial role in triggering somatic markers activated by secondary inducers [25].

Examining physiological reactions during the decision-making process is a crucial aspect of research design. For example, in a study that examined psychophysiological responses associated with pathological gambling (PG) during the *Iowa Gambling Task* (IGT), the PG group exhibited poorer performance on the task, lower anticipatory skin conductance responses, and decreased heart rate (HR) during deliberation on disadvantageous choices. Furthermore, the PG group displayed a decrease in HR after both wins and losses, suggesting the diminished reward sensitivity. In contrast, the control group showed an increased HR after wins. While reward and punishment sensitivity influenced both groups’ IGT performance and psychophysiological responses, the diminished anticipatory psychophysiological reactions in PG indicate the impaired risk assessment, emphasizing the importance of considering sensitivity levels in studies on responses to rewards and losses.

Some findings also suggest that engaging in gambling within a real-life setting leads to elevated salivary cortisol levels, accompanied by increased cardiovascular activity. These physiological responses may contribute to the development of gambling addiction (Meyer et al., 2000). Previous research on gambling behavior in the VR environment showed that participants express higher levels of arousal and immersion, as well as higher self-reported physical task workload in the game when playing in VR, compared to the same game played in the laboratory conditions [26]. Recent research has shown that even the 5-minute-long VR experience can induce emotional reaction as indicated by salivary α-amylase and heart rate variability [27]. In general, research findings support the notion of VR as an ecologically valid environment for exploring real-life behavior patterns.

It is evident that the rapid development of VR technology and its increasing integration into research provide researchers in various fields with a valuable tool for balancing experimental control and ecological validity. Previous research has shown that virtual environments offer realistic, life-like scenarios while maintaining the rigor and control necessary for experimental studies [28]. VR environments have several advantages over traditional experimental or questionnaire-based research designs for studying risky behavior. Firstly, they allow researchers to systematically investigate high-risk scenarios in a controlled laboratory setting, utilizing physiological assessments and conditions that are not possible in real-world environments [29]. Moreover, VR addresses ethical concerns by avoiding the need to expose participants to potentially unsafe situations. Finally, VR provides a valuable method for exploring the so-called "hot", affectively-salient processes, which are more difficult to capture than those related to traditional, often emotionally neutral, "cold" cognitive tasks [18].

### Current study

The primary focus of this study is to investigate patterns of risky decision-making in anticipation of the reward or punishment. Specifically, the aim is to explain the role of stable personality traits and environmental characteristics, particularly negative feedback, in risky decision-making. To address these questions, a VR environment was developed offering participants an opportunity to make more or less risky decisions while collecting points as a reward. The study is designed as a repeated measures experiment with two distinct time points—one before and the other after the introduction of negative feedback. It is expected that the proportion of riskier choices, the time taken to make decisions, the speed of movement, and the number of collected points, will differ between the two time periods.

The potential correlates of the participants’ behavior in the VR environment were explored within the rRST framework. Additionally, various indicators of physiological arousal were measured during the VR game: heart rate (HR), skin conductance (electrodermal activity–EDA), and respiratory rate (RR). The expectation is that risky decision-making is a multifaced process, encompassing previously learned patterns of behavior reflected in personality traits, different risk levels as the characteristics of situation, and physiological arousal that can determine specific responses. Specifically, it was anticipated that behavioral patterns in VR games would be influenced by characteristics of situation, as well as by stable personality traits, such as tendencies towards approach (BAS and Fight) and avoidance (BIS, Flight and Freeze). Additionally, we hypothesized that reactions in the VR environment will be associated with higher emotional engagement, as reflected by HR, EDA and RR.

The current study is specific and innovative in several ways. First, unlike some previous research where one or more personality traits were explored in relation to a potential threat [30,31], this research relates the rRST as a psychobiological model and a set of personality traits with both potential threat and actual punishment. Second, unlike similar repeated measures studies where VR was used merely to induce emotional reactions and stress response with participants who were basically passive [27,32], our study enabled comparisons of both physiological reactions and participants’ active behavior before and after negative feedback. And finally, our custom-made VR game was designed to provide a more real-life experience and choosing options in order to improve participants’ engagement and presence, as opposed to similar research that used, for example, platforms floating in empty space [30] or blowing up a balloon [22]. It has also enabled the collection of specific data within the VR environment itself and relating it to specific locations in the game, which is not possible when similar pre-made games are used in research.

## Method

### Participants

To ensure the heterogeneity of participants regarding their previous experience with computers, age, and gender, the sample consisted of 52 students from the University of Novi Sad, Serbia: 27 students from the Faculty of Philosophy, and 25 students from the Faculty of Technical Sciences. The power analysis conducted in G*Power [33] showed that this sample is sufficiently large to detect differences using the paired samples t-test at 0.05 α level, and expected effect size of 0.55. Most participants from the Faculty of Philosophy were females (85.2%) while most participants from the Faculty of Technical Sciences were males (60.0%). The whole sample consisted of 33 females (63.5%) and 19 males (36.5%). The mean age of participants in the whole sample was 23.56 (SD = 7.76). Participants from the Faculty of Technical Sciences (M = 28.40, SD = 8.99) were statistically significantly older (t(50) = -5.56, p < .001) than participants from the Faculty of Philosophy (M = 19.40, SD = 0.99). These age differences were anticipated since all participants from the Faculty of Philosophy were in the first year of bachelor’s degree studies, while participants from the Faculty of Technical Sciences were mostly in the third and fifth years of Bachelor’s degree studies.

The research was approved by the Ethical Committee of the Faculty of Philosophy in Novi Sad (#02-374/15). All participants provided informed consent for participating in the research using a custom-made online portal specifically developed for the project within which the research was conducted. Only participants who provided informed consent and completed the questionnaire were invited to take part in the VR experiment. Questionnaire and VR data were linked using unique codes assigned to participants upon confirming their willingness to participate. No personal data was recorded apart from contact email addresses, which were removed from the database after the completion of the VR experiment. All data were analyzed anonymously. The recruitment period started on December 21, 2022, and ended on April 4, 2023.

### Psychological measures

In the first phase of the research, all participants completed the *Reinforcement Sensitivity Questionnaire* (RSQ) [34] in order to assess their stable personality traits. RSQ is based on the *Revised Reinforcement Sensitivity Theory* [9]. It contains 29 items on five scales: *Behavioral Inhibition System* (BIS) (7 items, α = .75), *Behavioral Activation System* (BAS) (6 items, α = .75), *Fight*, *Flight* and *Freeze* system (with 5 items each, α = .74, .52, .75, respectively). Items are rated on a four-point Likert scale (from 1 = *completely disagree* to 4 = *completely agree*).

### VR environment

**Fig 1:**
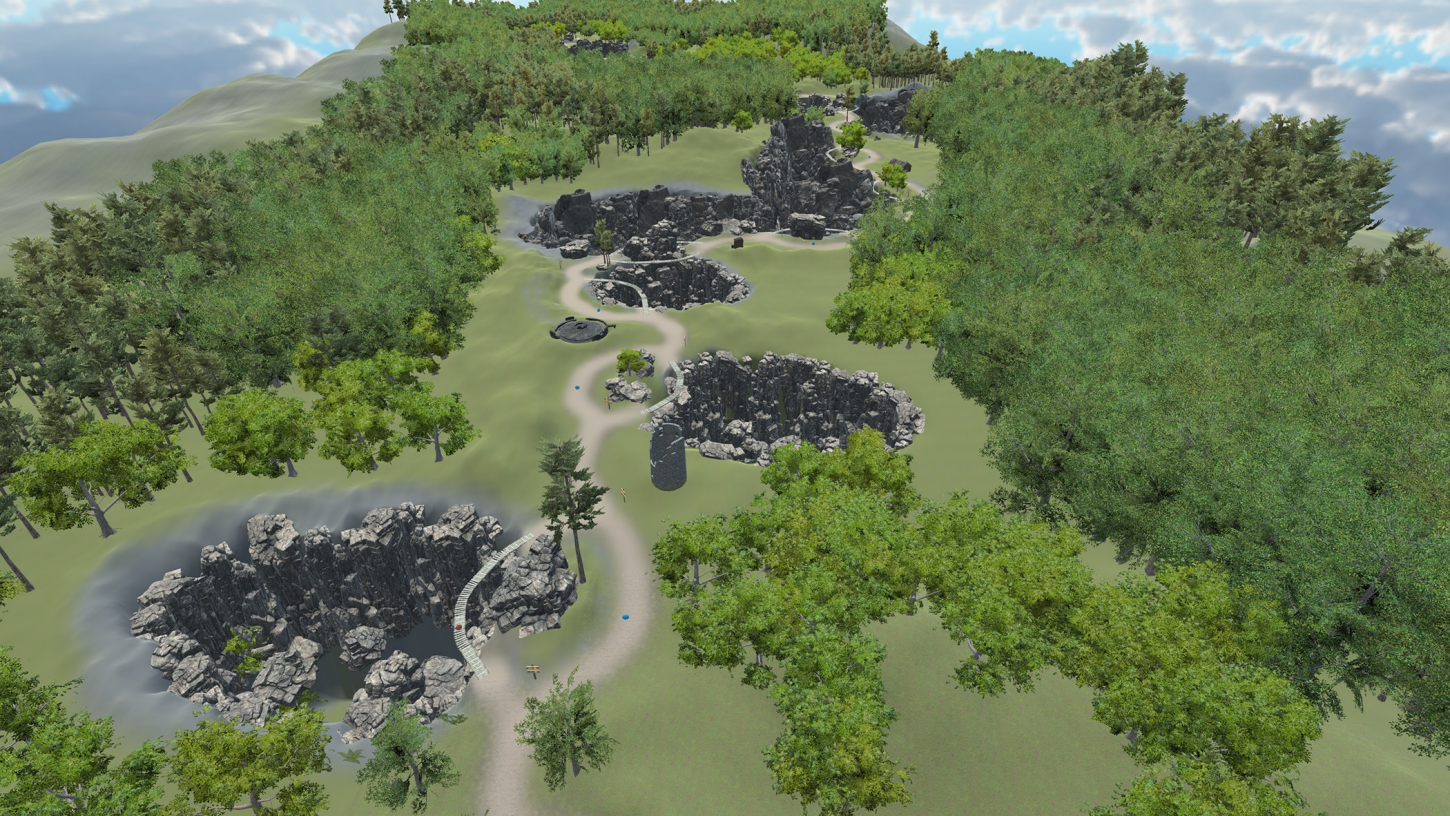
Aerial overview of the path in the VR environment.

**Fig 2:**
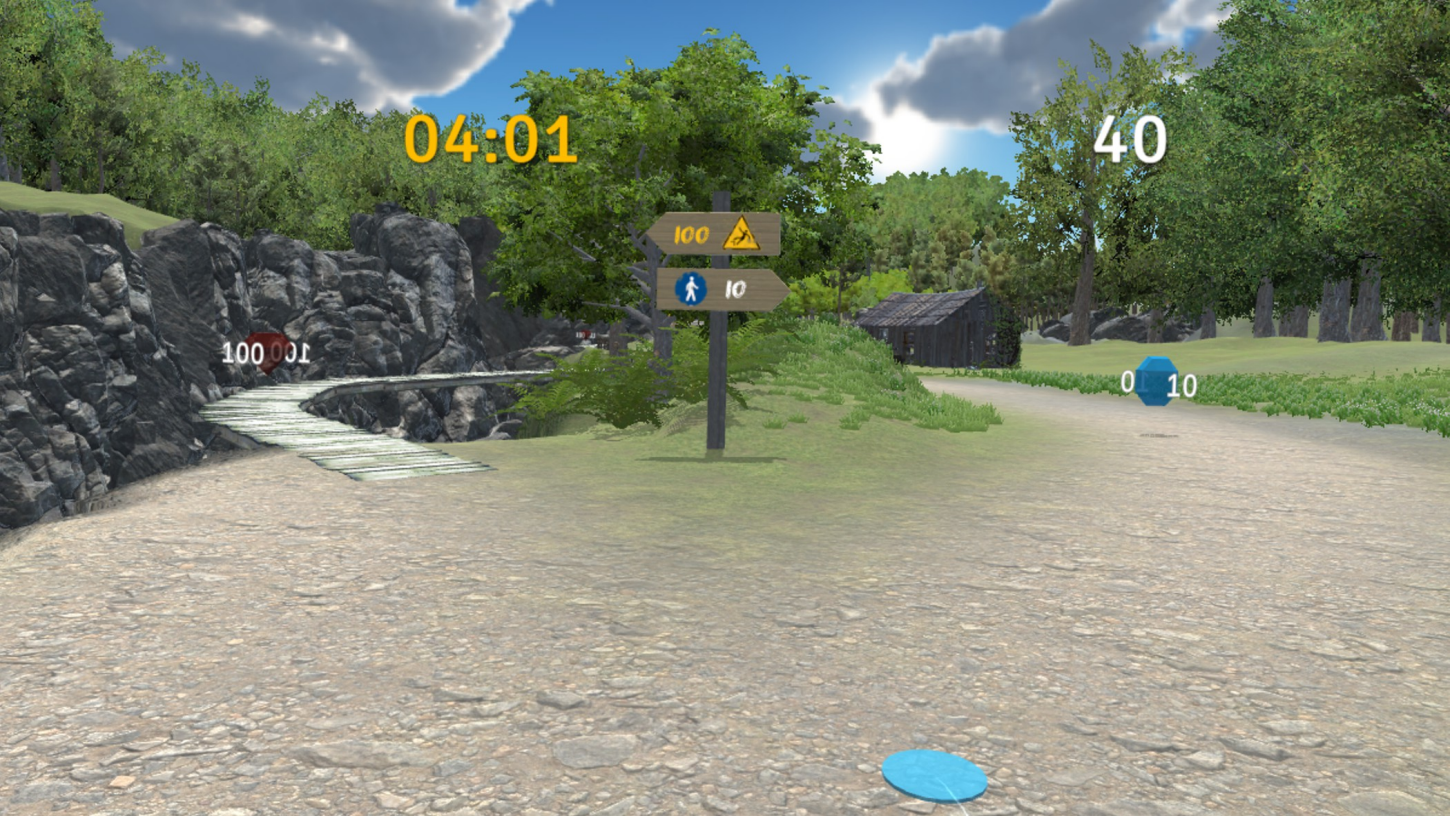
Example of the bifurcation with one safe and one risky path.

**Fig 3:**
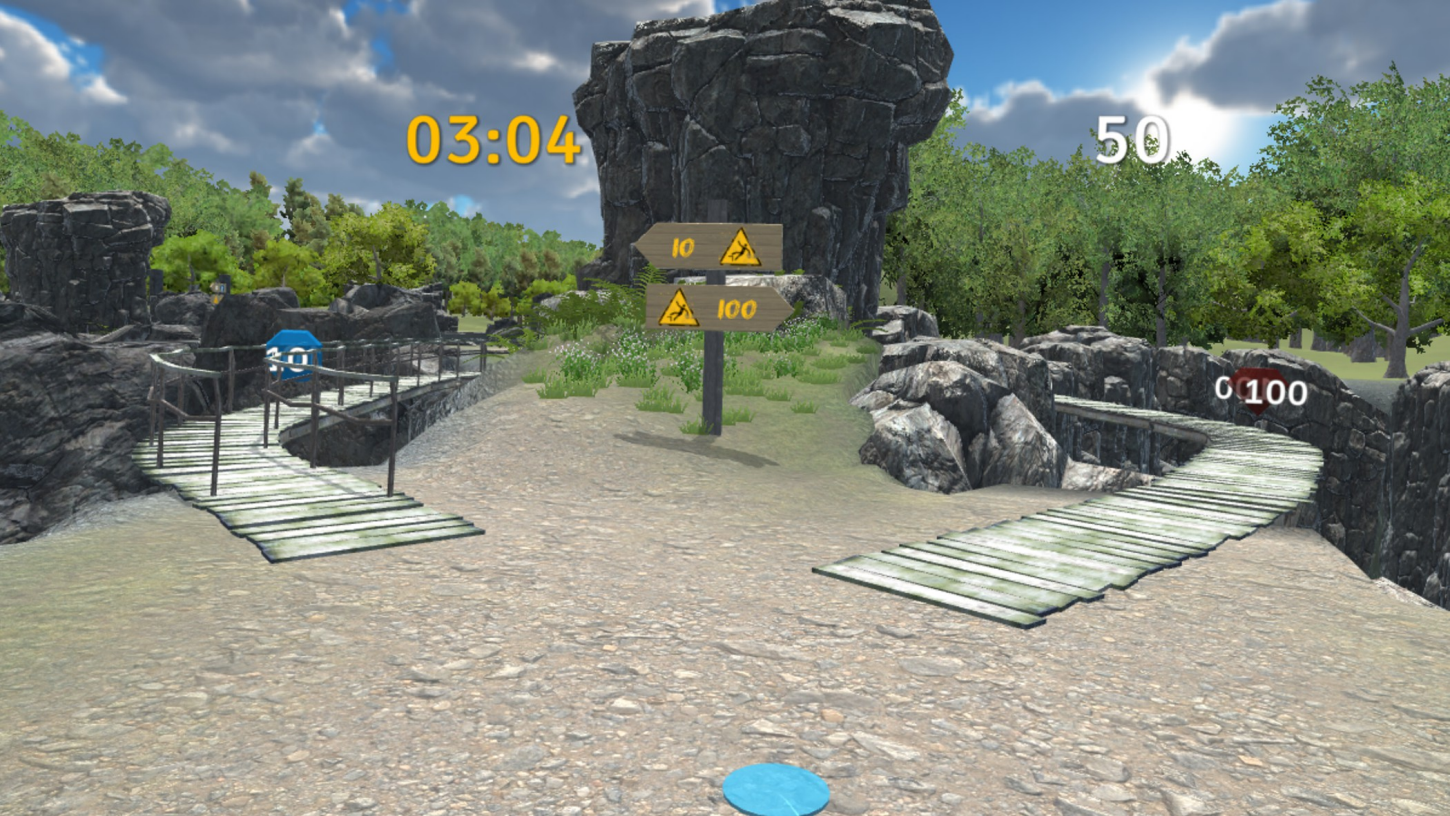
Example of the bifurcation with two risky paths of different values.

In the second phase of the study, participants were engaged in the VR experiment. For this purpose, a custom VR environment was created in *Unity* version 2021.3.6f1. We used the Oculus Rift S VR headset with a Lenovo Legion Y530 laptop, which features an Intel® Core™ i7-8750H processor running at 2.20 GHz, a GeForce GTX 1050 Ti graphics card with 4GB GDDR5, and 32 GB of RAM. The environment was designed as a 5-minute game in which participants had to navigate a path through virtual nature and collect diamonds (Fig 1). Along their journey towards the end of the path, they encountered 20 bifurcations where they had to choose between a safer dirt road and a riskier path across a wooden bridge. Safer roads yielded 10 points, while crossing the bridge was awarded 100 points (Fig 2). Three bifurcations, located at points 5, 10, and 16, featured two bridge crossings, one worth 10 points and the other 100 (Fig 3).

The initial instruction for participants was to freely choose between paths, and they were informed that the bridges could only collapse randomly. However, the game was designed so that only the bridges at point 10 collapsed for each participant, regardless of whether he or she had chosen a 10-point or a 100-point one. The collapse of the bridge is expected to be negative feedback in the process of decision-making. Following the bridge collapse, participants could continue playing the game and collecting diamonds until the time expired.

To mitigate the risk of potential nausea, participants did not move continuously along the path. Instead, they were moving by teleporting, i.e., by clicking on a distant point to transition to their next location. By using the teleportation mode of movement, it was also possible to record the number of steps participants took during the game.

Four measures were generated within the VR environment both for the period before and after the collapse of the bridge. In the Results section, all variables for the first period have the suffix “Bf”, while all the variables for the second period have the suffix "Af”:

*Time* – Elapsed game time in seconds;

*Steps* – Number of steps, i.e., clicks of the controller trigger button;

*Score* – Average score, i.e., the mean value of collected diamonds;

*DecT* – Median time taken to make a decision at each bifurcation in seconds.

**Fig 4:**
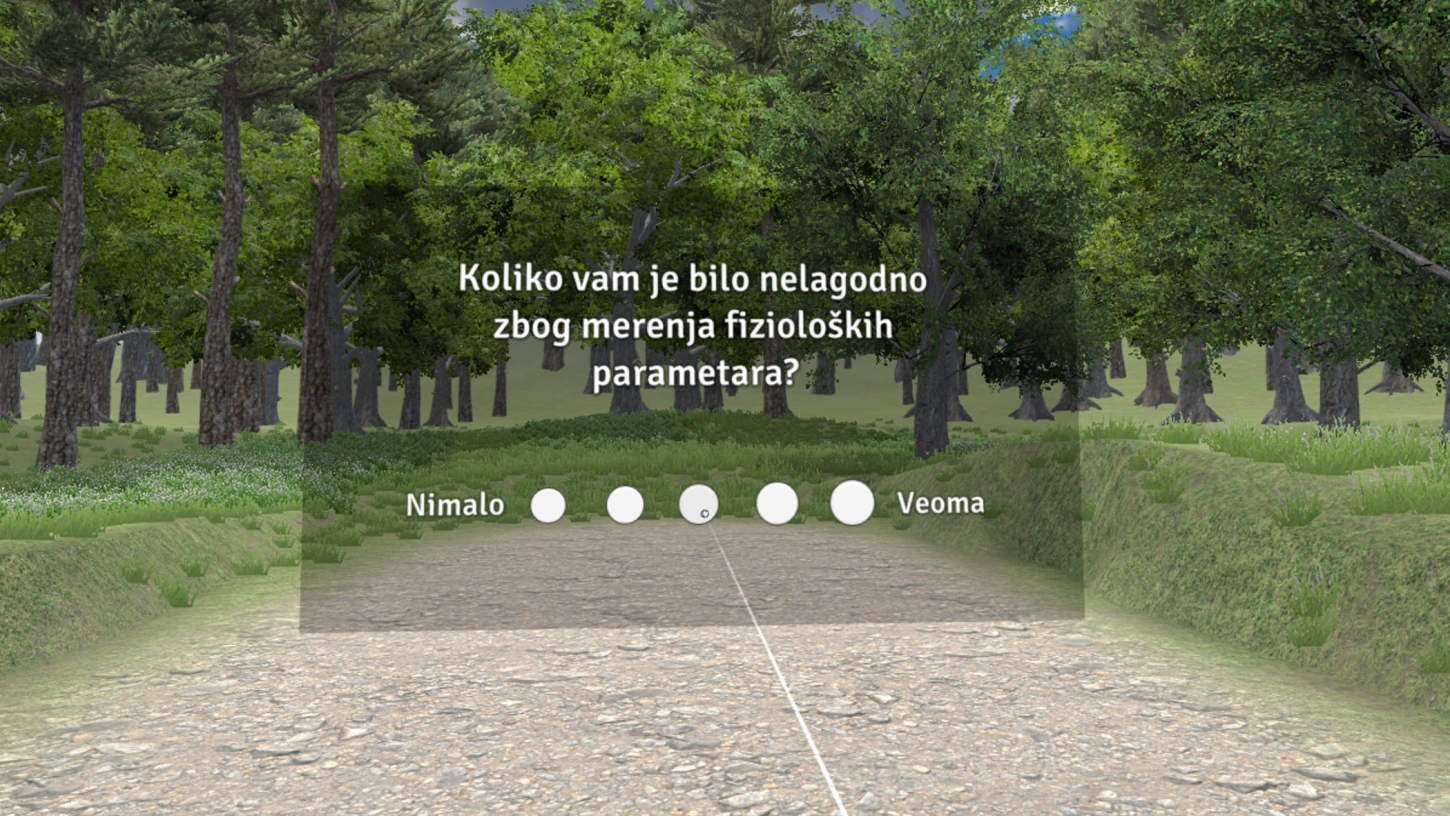
Example of the questionnaire item within the VR environment (“How uncomfortable you were with the measurement of physiological parameters” in Serbian).

In addition to these measures, all participants completed a brief questionnaire at the end of the game, while still immersed in the VR environment (Fig 4). The questionnaire comprised five 5-point Likert-type questions, addressing whether they enjoyed the game, felt discomfort, experienced distress when the bridge collapsed, felt upset about losing all the points, and their frequency of playing videogames. After the game completion, all participants were invited to a short debriefing session with an experienced psychologist to receive feedback on the research design and provide them with an opportunity to discuss their feelings, emotional arousal, and any potential distress.

### Physiological measures

Three significant signals were measured. EDA (electrodermal activity), ECG (electrocardiography) and respiratory rate were used to measure respiratory rate amplitude, median heart rate, and skin conductance level. EDA measurements have been done with two electrodes placed on the left-hand index and middle fingers. The ground measurement electrode has been placed on the middle finger, whilst the second electrode was placed on the index finger. For ECG signal acquisition, three electrodes were used as well, forming the Einthoven’s triangle by two of them being attached to the right and left hand, creating a differential input, and the last electrode was used for noise reduction, by being attached to the right leg (DRL electrode-driven right leg). To improve the conductivity of the electrodes, an abrasive pad was used for thorough skin cleaning, and the conductive gel was applied prior to adhering the electrodes. The respiratory rate was measured using the breathing belt. The transducer was used to converse the mechanical energy associated with the expansion and contraction of the chest during breathing into an electrical signal.

**Fig 5:**
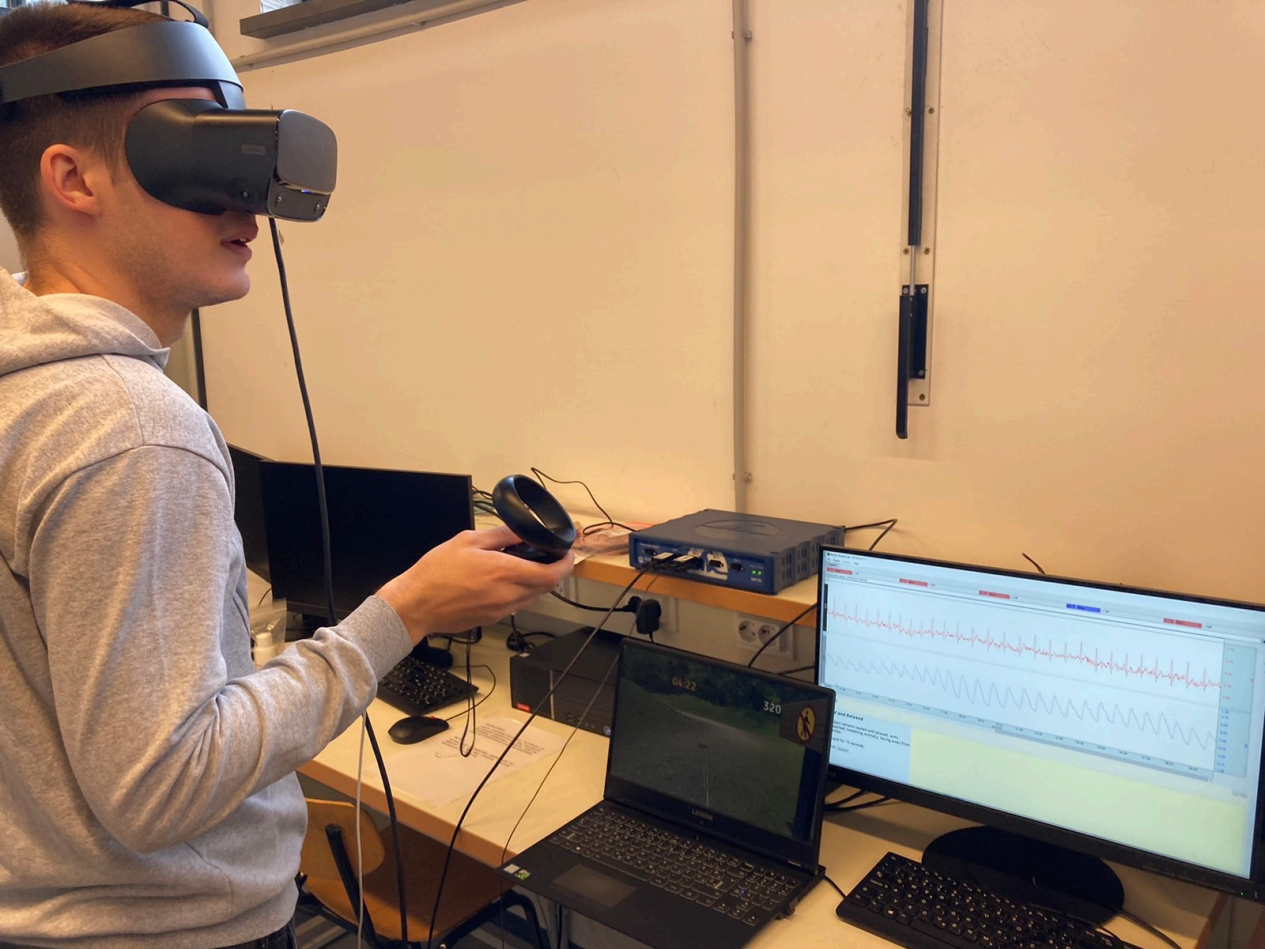
One of the participants in the experimental environment.

The measurements were carried out on subjects during the VR game, using the BIOPAC MP36 system. Each measurement was started when the examinee pressed the start button within the game so that collected electrophysiological signals can be matched with the variables from the VR environment. Custom MATLAB and Python scripts were used for signal processing and feature extraction, whilst GraphPad PRISM was used for data visualization. The image of a participants engaged in the VR game and the equipment used in the experiment is shown in Fig 5. A schematic image of a participant, illustrating the precise positions of all electrodes is available in the supplementary material deposited in the OSF repository at https://osf.io/5grmk/.

Six electrophysiological measures were calculated at two-time intervals, during a minute before the collapse of the bridge in the VR environment and during a minute after the collapse. In the Results section, all variables for the first period have the suffix “Bf”, while all the variables for the second period have the suffix "Af”:

*HRMdn* – Median heart rate, defined as the median value of heart rate in a specific, previously defined interval;

*HRSpan* – Heart rate span, defined as the difference between the maximum and minimum values over all calculated heart rate values in a specific interval;

*SCLvl* – Skin conductance level, defined as the mean value of the electrodermal activity in a specific time interval, after denoising and detrending processes had been applied;

*SCRAmp* – Skin conductance rate amplitude, defined as the difference between maximum values of the electrodermal activity signal and the previously defined skin conductance level;

*RR* – Respiratory rate, defined as the number of breaths or respiratory cycles one takes in a specific time frame (most commonly 60 seconds);

*RRAmp* – Respiratory rate amplitude or respiratory amplitude, defined as the difference between the maximum and the minimum values of the recorded respiratory signal.

**Fig 6:**
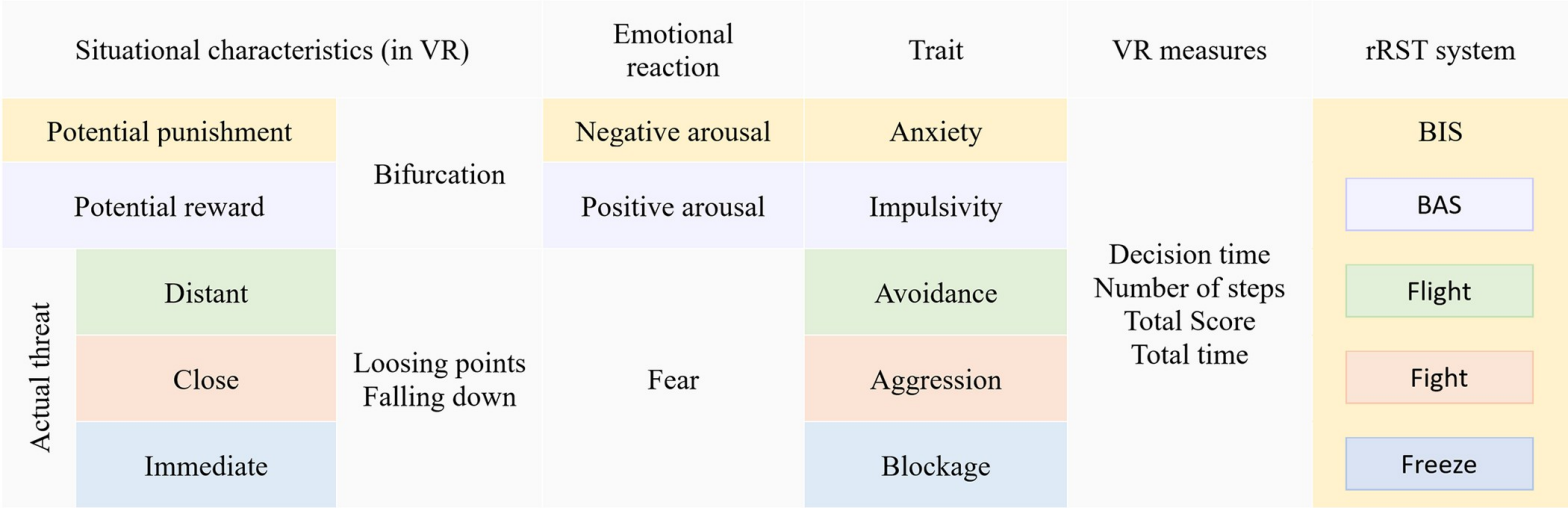
Measures of individual differences and characteristics of situation assessed before and after the negative feedback.

Fig 6 illustrates the general model of the experiment, encompassing situational characteristics, emotional reactions, generated VR variables, and personality traits. Situational characteristics refer to VR stimuli designed to provoke the experience of potential and real threat or reward, such as bridges and reward points at bifurcations. Anticipated emotional reactions to these stimuli were fear, and positive and negative arousal. These emotional reactions can be associated with real-life risky situations and accompanying behaviors: anxiety, related to the BIS, impulsivity, related to the BAS, as well as flight, fight or freeze behavior.

### Data analysis

Log files generated within the VR environment were processed and prepared for the analysis using the custom-made Python scripts. Data manipulation, curation, and preparation was carried out using the Python *pandas* package [35], and all graphs were generated using the *Plotly* [36] and *Matplotlib* [37] packages.

Electrophysiological signals described in the previous section were processed in MATLAB. Moreover, the signals were denoised and adequately filtered in order to extract previously defined electrophysiological features. Detailed code and procedures for the filtering process, signal processing, and calculation of physiological measures used in the experiment are explained in documents available in the OSF repository at https://osf.io/5grmk/.

All statistical analyses on the final data matrix containing the variables from all three categories (VR, psychological, and physiological) were performed in SPSS v25 [38]. Given that some data did not meet the homogeneity of variance assumption and/or didn’t exhibit a normal distribution, we opted for appropriate nonparametric tests, using the Welch test and the Mann–Whitney U test instead of the classic t-test, as they are known to be robust to violations of these assumptions and provide a more reliable estimate of the differences between groups. For comparing multiple groups, we used the Kruskal-Wallis ANOVA as an alternative to parametric ANOVA, while Goodman and Kruskal’s gamma and Spearman’s Rho coefficients of correlation were applied when the assumption of normality was violated or when the variables were ordinal in nature, such as the responses to the VR questionnaire items. Anonymized dataset is deposited in the OSF repository at https://osf.io/5grmk/.

## Results

The results are organized into four sections. The first contains basic descriptive statistics for all measures. The second contains the analysis of differences in VR measures a minute before and after the collapse of the bridge as negative feedback. The third contains the analyses of selected results related to the responses to the short questionnaire given while the participants were still immersed in the VR environment. Finally, the fourth section presents the results of the regression analysis that correlates VR measures with physiological measures and personality traits.

### Descriptive statistics

Tables 1–3 show the basic descriptive statistics for psychological, VR, and physiological measures respectively. Descriptive statistics were calculated mainly to check for the normality of distribution, but also to examine the potential discriminability of each variable based on its variability. All variables have the values of skewness and kurtosis within the acceptable ranges [39], except for the amplitude of skin conductance rate, due to several outliers. The variability in all measures indicates sufficient differences among individuals, and hence their possible discriminative capacity. For example, the average time to make a decision on bifurcations varied from less than a second to more than four seconds. Sample sizes vary across tables because not all participants had the values for physiological measurements due to poor signal quality.

**Table 1:** Descriptive statistics for the psychological measures used in the experiment.

Trait	N	M	Min	Max	SD	Skewness	Kurtosis
BIS	52	17.42	7	27	4.18	0.092	-0.244
BAS	52	17.17	9	24	3.43	-0.098	0.121
Fight	52	14.57	8	21	3.27	-0.196	-0.419
Flight	52	14.44	8	20	2.70	0.115	-0.516
Freeze	52	10.34	5	19	3.38	0.238	-0.434

**Table 2:** Descriptive statistics for the VR measures used in the experiment.

Measure	N	M	Min	Max	SD	Skewness	Kurtosis
TimeBf	52	120.60	57.99	218.55	36.84	0.674	0.053
TimeAf	52	97.12	51.62	175.92	29.22	0.685	0.083
StepsBf	52	78.94	43.00	156.00	25.26	1.280	1.875
StepsAf	52	92.40	43.00	191.00	35.46	1.044	1.011
ScoreBf	52	62.77	19.00	100.00	19.76	0.057	-0.746
ScoreAf	52	58.38	10.00	100.00	24.69	0.152	-0.487
DecTBf	52	2.38	0.38	4.60	0.89	0.041	-0.099
DecTAf	52	1.46	0.26	3.84	0.86	0.754	0.077

*Note*: *Time*–Elapsed game time in seconds; *Steps*–Number of steps (trigger presses); *Score*–Average score, i.e., value of collected diamonds; *DecT*–Median time in seconds taken to make a decision at each bifurcation; “*Bf*” (suffix)–Before the bridge collapse; “*Af*” (suffix)–After the bridge collapse.

**Table 3:** Descriptive statistics for the physiological measures used in the experiment.

Measure	N	M	Min	Max	SD	Skewness	Kurtosis
HRMdnBf	47	93.36	71.71	112.39	11.76	-0.36	-1.06
HRMdnAf	47	93.26	71.96	110.95	10.58	-0.36	-0.61
HRSpanBf	47	43.10	10.22	64.78	17.89	-0.46	-1.42
HRSpanAf	47	46.96	17.84	62.19	15.10	-0.67	-1.18
SCLvlBf	47	15.32	0.08	61.16	13.23	0.92	1.48
SCLvlAf	47	16.28	0.15	65.57	14.38	1.04	1.57
SCRAmpBf	45	0.31	-0.56	3.70	0.68	3.10	13.52
SCRAmpAf	46	0.18	-3.37	3.83	1.12	-0.09	4.58
RRBf	47	15.75	12.32	21.01	2.32	0.61	-0.44
RRAf	47	15.49	11.15	19.26	1.70	0.02	0.23
RRAmpBf	47	6.19	0.27	15.56	5.11	0.53	-1.33
RRAmpAf	47	8.12	0.66	16.63	5.61	0.07	-1.68

*Note*: *HRMdn*–Median heart rate; *HRSpan*–Heart rate span; *SCLvl*–Skin conductance level; *SCRAmp*–Skin conductance rate amplitude; *RR*–Respiratory rate; *RRAmp*–Respiratory rate amplitude; “*Bf*” (suffix)–Before the bridge collapse; “*Af*” (suffix)–After the bridge collapse.

### Analyses of differences

Table 4 shows the values of the t-tests for paired samples and their corresponding significance and effect sizes for VR measures before and after the collapse of the bridge. On average, all VR measures have shown a decrease after the negative feedback, except for the number of steps, which increased significantly. Furthermore, all differences are statically significant, except for the average score, which has also decreased, but only approaching the significance threshold. Nevertheless, it is clear that after the collapse of the bridge participants have significantly changed their behavioral pattern. They were more focused on finishing the game more rapidly, making faster and more precise movements by making more steps, spent less time deciding which path to go, and more often collecting less valuable diamonds on safer roads. Although these differences may partially be attributed to the practice and experience they were gaining towards the end of the game, this factor could not influence the decision time nor the decision on which path to choose. In relation to participants’ previous experience, no significant correlations were found between the frequency of playing videogames and any of the variables given in Table 4.

**Table 4:** Statistical significance of differences in VR measures before and after the collapse of the bridge.

Measure	Mean Bf	Mean Af	Δ Mean	t	df	p	d
Time	120.60	97.12	23.48	6.005	51	< .001	0.833
Steps	78.94	92.40	-13.46	-4.023	51	< .001	-0.558
Score	62.77	58.38	4.39	1.953	51	.056	0.271
DecT	2.38	1.46	0.92	8.105	51	< .001	1.124

*Note*: *Time*–Elapsed game time in seconds; *Steps*–Number of steps; *Score*–Average score, i.e., value of collected diamonds; *DecT*–Median time in seconds taken to make a decision at each bifurcation; Bf–Before negative feedback; Af–After negative feedback; d–Effect size as Cohen’s d.

Regarding gender differences, it is noteworthy that females were more inclined to choose bridge crossings, i.e., more valuable and riskier paths. This difference is evident in average score differences both before and after the bridge collapse. Welsh’s t-test for the score difference before was t(48.04) = -3.53; p < .001, while for the average score after negative feedback was t(48.43) = -3.03; p = .004. However, this does not imply that females were more impulsive or hasty when choosing the path. On the contrary, although there was no difference in time or decision time before the bridge collapse, the time taken to walk along the path after the collapse was significantly higher for females: t(49.63) = -2.93; p = .005, as well as the time taken to make a decision: t(48.84) = -2.17; p = .035.

**Fig 7:**
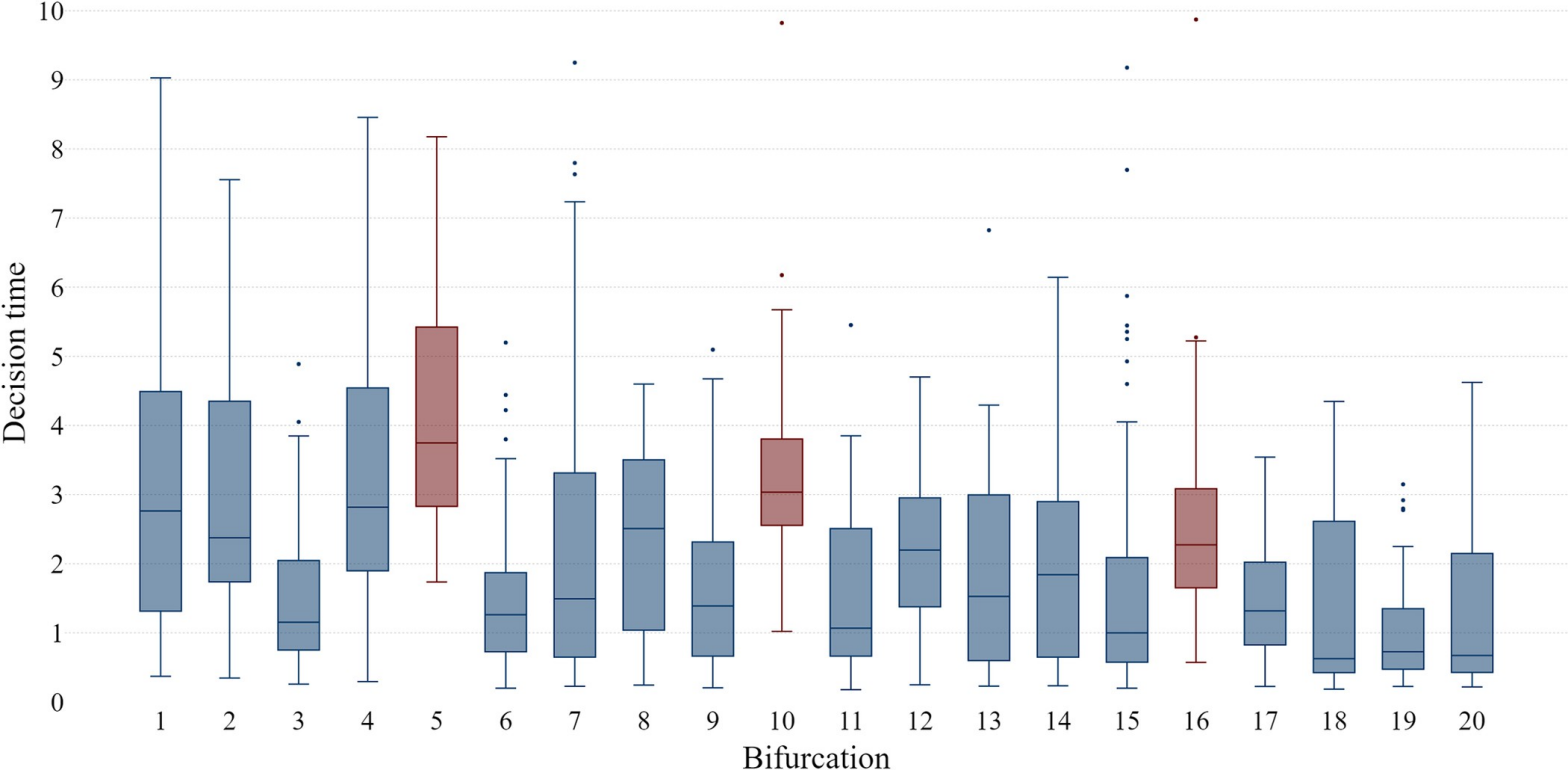
Median and quartile range of time taken to make a decision at each bifurcation (bifurcations with two bridges are marked in red).

In order to further explore the ecological validity of the decision time as a measure of behavior in VR, its values were compared across different locations in the game. Fig 7 shows medians and quartile ranges for decision times at each bifurcation. Two patterns are noticeable. The first is that the decision time is constantly decreasing, particularly after the collapse of the bridge, when the variability of decision time among participants also decreased. The second pattern reveals higher decision times on bifurcations with two bridges (marked in red) which supports the validity of this VR measure. Having the information that bridges can randomly fall, regardless of the number of points they grant, participants were spending more time deciding which of the two risky paths to choose when compared to bifurcations with only one risky path. Basically, these are the two slightly different decisions that could be related to risky choice framing vs. goal framing. The first implies the choice between the safe and risky path, and the second between two gains with the same possibility for loss.

**Fig 8:**
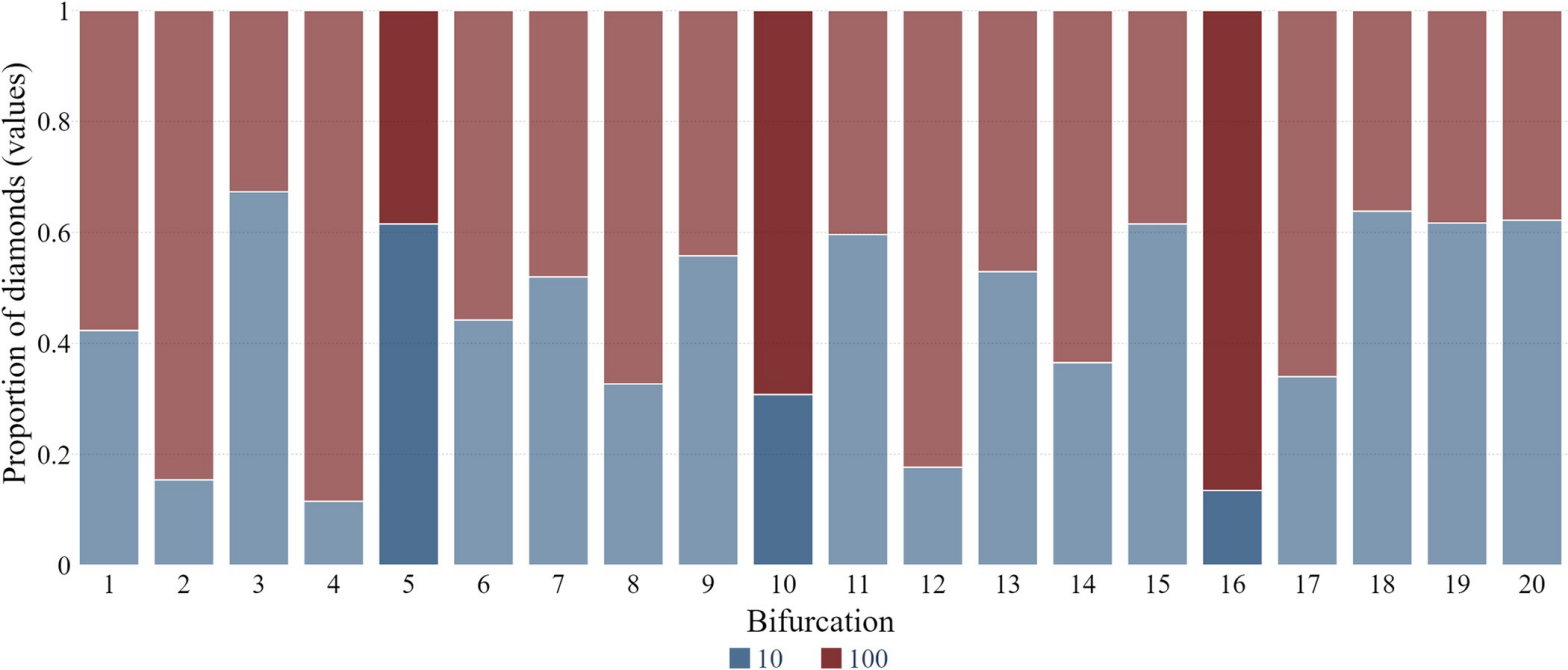
The proportion of diamonds of different values collected at each bifurcation (bifurcations with two bridges are marked with darker colors).

We have shown that participants were spending less and less time deciding which path to choose as the game progressed, although they still had longer decision times at bifurcations with two bridges. It is also worth examining the typical outcomes of these decisions. Fig 8 shows the proportion of diamonds of different values the participants collected at each location. Although it may seem there is a random strategy of alternating between the safe 10-point and risky 100-point paths at successive bifurcations, two noticeable patterns emerge. The first is the difference between a few initial and a few final bifurcations. At the beginning of the game, participants were more frequently choosing riskier paths, while at the very end of the game they were less willing to risk losing all the points. The last three bifurcations have obviously contributed the most to the lower average scores after the collapse of the bridge.

The second pattern noticeable in Fig 8 concerns double-risky bifurcations. At the first bifurcation of this type, most of the participants have chosen the bridge they probably estimated as safer because it yielded 10 points. Conversely, at the point of collapse most of them have chosen the 100-points bridge. Finally, although the majority of participants have chosen the more valuable path at 10^th^ bifurcation (resulting in the loss of all points), the absolute majority of them have also chosen the more valuable path at the last double-risky bifurcation. It seems that the participants have shifted from the risky choice framing to goal framing, being less willing to risk at the beginning of the game, while choosing the more rewarding risky path among the two as the game was approaching its end.

Table 5 shows the values of the t-tests for paired samples and their corresponding significance for physiological measures before and after the collapse of the bridge. In order to control for the possible outliers due to the higher arousal at the very beginning and the very end of the game, measures were calculated for the periods of one minute preceding and one minute following the collapse of the bridge. Nevertheless, there was no significance in the median heart rate, although the heart rate showed a difference very near to the significance threshold. The participants were obviously generally very aroused due to the experimental situation itself, at least as indicated by their heart rate. The only two measures that changed significantly between the two time periods were the level of skin conductance and the respiratory rate amplitude, both increasing significantly following the fall from the bridge. The latter difference is particularly prominent.

**Table 5:** Statistical significance of differences in physiological measures before and after the fall of the bridge.

Measure	Mean Bf	Mean Af	Δ Mean	t	df	p	d
HRMdn	93.36	93.26	0.10	0.11	46	.910	0.017
HRSpan	43.10	46.96	-3.86	-1.91	46	.063	-0.278
SCLvl	15.32	16.28	-0.96	-2.61	46	.012	-0.381
SCRAmp	0.31	0.18	0.13	0.71	44	.482	0.106
RR	15.75	15.49	0.25	0.78	46	.438	0.114
RRAmp	6.19	8.12	-1.93	-4.01	46	< .001	-0.585

*Note*: *HRMdn*–Median heart rate; *HRSpan*–Heart rate span; *SCLvl*–Skin conductance level; *SCRAmp*–Skin conductance rate amplitude; *RR*–Respiratory rate; *RRAmp*–Respiratory rate amplitude; Bf–Before negative feedback; Af–After negative feedback; d–Effect size as Cohen’s d.

### Validation of the subjective estimate of agitation

In order to estimate the validity of physiological measures, participants’ response to the question related to the subjective estimate of agitation due to the collapse of the bridge was compared to objective measures of excitement. Table 6 shows gender differences in measures previously detected having the highest effect sizes: heart rate span, skin conductance level, and respiratory rate amplitude. All physiological measures were higher for females, but only the difference in the heart rate span before the bridge collapse is statistically significant. Additionally, skin conductance level is approaching the level of statistical significance, both for the period before and after the collapse. This indicates that participants reacted differently to the collapse of the bridge. In the group of males, the difference in heart rate span is statistically significant (t(15) = -3.09, p < .001), while in the group of females it is not (t(30) = 0.32, p = .75). Exactly the opposite results have been found for the skin conductance level and respiratory rate amplitude. The change in SCLvl is statistically significant for the females (t(30) = -2.13, p = .04), and not for the males (t(15) = -1.62, p = .13). The change in RRAmp is also statistically significant for the females (t(30) = -4.50, p < .01), and not for the males (t(15) = -0.87, p = .39).

**Table 6:** Gender differences in selected physiological measures.

Measure	Males			Females			Welch’s t-test	df	p
	N	M	SD	N	M	SD			
HRSpanBf	16	34.23	20.15	31	47.68	14.97	-2.36	23.79	.027
HRSpanAf	16	46.71	13.35	31	47.09	16.14	-0.09	35.95	.932
SCLvlBf	16	8.92	17.49	31	18.62	9.09	-2.08	19.28	.051
SCLvlAf	16	9.62	18.17	31	19.71	10.79	-2.04	20.61	.054
RRAmpBf	16	6.13	6.06	31	6.22	4.65	-0.05	24.39	.961
RRAmpAf	16	6.86	5.99	31	8.77	5.40	-1.07	27.78	.295

*Note*: *HRSpan*–Heart rate span; *SCLvl*–Skin conductance level; *RRAmp*–Respiratory rate amplitude; “*Bf*” (suffix)–Before the bridge collapse; “*Af*” (suffix)–After the bridge collapse.

**Fig 9:**
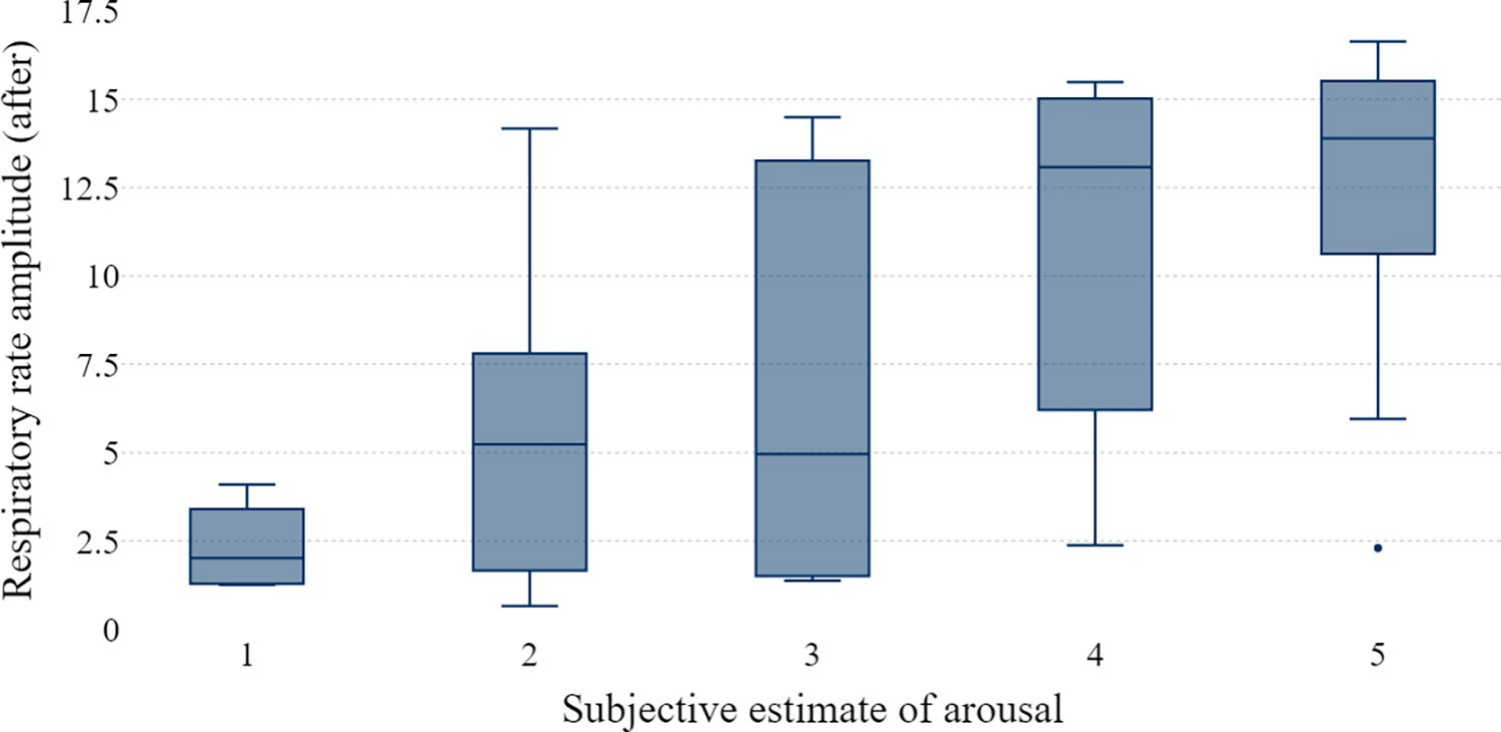
Correspondence between the respiratory rate amplitude and the subjective estimation of the arousal due to the fall of the bridge.

In the next step, differences in selected measures of physical excitement are tested against the responses to the question of how upset they were due to the fall of the bridge using the Kruskal-Wallis ANOVA. Median heart rate was not related to the participant’s estimate of agitation (H(4, 48) = 2.79; p = .594), nor was the skin conductance level (H(4, 48) = 3.52; p = .474). However, the correspondence between the estimate of agitation and the respiratory rate amplitude was significant: H(4, 48) = 15.50; p = .004. In general, participants who have stated they were more upset due to the collapse of the bridge had higher respiratory rate amplitudes in the period of a minute following the negative feedback (Fig 9).

Finally, it is also worth mentioning that participants’ perceived realism of the VR environment has significant correlations with both their estimate of arousal due to the fall of the bridge (G = .42, p < .001) and the loss of all points (G = .42, p < .001). On the other hand, the correlation between the perceived realism is negatively correlated with the frequency of playing computer games (-.29, p = .02). Finally, females have generally perceived the VR environment as more realistic than males (U = 175, p = .008).

### Correlation and regression analyses

Spearman’s rank correlation coefficients for all measures used in the experiment are presented in Tables A, B, and C of the Supplementary Material. BAS was significantly negatively correlated with the time taken to complete the game, both before (ρ = -0.31, p = .023) and especially after receiving negative feedback (ρ = -0.42, p = .002). Participants with higher BAS scores exhibited more impulsive approach behaviors when confronted with risky scenarios, taking less time to fully weigh potential risks. In contrast, participants who were more prone to avoidance behaviors reacted to negative feedback by taking more time to complete the game, as reflected in the positive correlation between elapsed time after the bridge collapse and both BIS (ρ = 0.29, p = .041) and Freeze (ρ = 0.28, p = .043). However, these results should be interpreted with caution, as the practical significance of these coefficients is relatively limited. The strongest correlation was observed between Freeze and the number of steps taken before negative feedback (ρ = 0.46, p < .001), indicating that higher Freeze scores are linked to more cautious and fragmented movements. However, this behavior was not observed after the bridge collapse. Median heart rate did not show significant correlations with any VR or psychological measure, but heart rate span was positively correlated with Flight before (ρ = 0.36, p = 0.01) and Freeze after the negative feedback (ρ = 0.31, p = 0.04). Lastly, respiratory rate amplitude was positively correlated with BIS (ρ = 0.29, p = 0.049) and Freeze (ρ = 0.34, p = 0.02) before, and the number of steps, both before (ρ = 0.38, p < 0.01) and after negative feedback (ρ = 0.40, p < 0.01).

Regression analyses were performed for each VR measure separately before and after the collapse of the bridge in order to detect their relationships with personality traits. Apart from the psychological variables, respiratory rate amplitude was also used as a predictor since it was the variable that indicated the most prominent physiological change after the negative feedback. Of all the VR measures, only the number of steps yielded statistically significant models—before (R = .572, R^2^_ADJ_ = .227, RMSE = 23.12, F(6, 40) = 3.25, p = .011) and after the collapse of the bridge (R = .527, R^2^_ADJ_ = .170, RMSE = 33.21, F(6, 40) = 2.57, p = .034). These results may be attributed to the fact that the only tangible physical and controlling activity participants needed to execute during the VR game was using the trigger button, i.e., initiating teleportation steps. Results for the models where the number of steps before and after the collapse of the bridge is used as criteria are shown in Table 7.

**Table 7:** Results of the regression analysis with the number of steps as a criterion and personality traits and respiratory rate amplitude as predictors.

Model		B	SE	β	t	p
StepsBf	BIS	-0.669	1.049	-.108	-0.638	.527
	BAS	0.786	1.122	.103	0.7	.488
	Fight	-2.563	1.092	-.33	-2.347	.024
	Flight	-2.45	1.490	-.244	-1.645	.108
	Freeze	3.409	1.234	.431	2.762	.009
	RRAmpBf	1.42	0.688	.276	2.065	.045
StepsAf	BIS	-0.409	1.521	-.047	-0.269	.790
	BAS	2.095	1.646	.198	1.273	.210
	Fight	-3.103	1.570	-.288	-1.977	.055
	Flight	-2.981	2.164	-.214	-1.378	.176
	Freeze	3.382	1.777	.309	1.903	.064
	RRAmpAf	2.187	0.961	.337	2.276	.028

*Note*: B–unstandardized coefficient; β –standardized coefficient; SE–standard error; StepsBf–Number of steps taken before the bridge collapse; StepsAf–Number of steps taken after the bridge collapse; RRAmpBf–Respiratory rate amplitude before the bridge collapse; RRAmpAf–Respiratory rate amplitude after the bridge collapse.

The results of the regression analyses show that personality traits are significantly related to the behavior in the VR environment in the respect that participants who made more steps, i.e., who were clicking the trigger button more frequently, have lower scores on Fight and higher scores on Freeze. The latter may be explained in the sense that participants with higher scores on Freeze tended to move more carefully and gradually, taking shorter steps. The effects of personality traits, however, are not significant after the collapse of the bridge, i.e., after the negative feedback. Additionally, emotional arousal measured by the respiratory rate amplitude is significant in both models, but plays a more important role after the collapse of the bridge. This indicates that behavior in the VR environment is more determined by the personality traits before the negative feedback, and by the characteristics of the task after the negative feedback. Participants were more aroused after the bridge fall and generally were spending less time deciding which path to follow, more often choosing safer paths, but at the same time being more willing to take the risk at double-risky bifurcations.

## Discussion

In the present study, the process of decision-making in risk situations, specially tailored within the VR environment, was analyzed through experimental design. The primary objective was to examine how individuals make risky decisions when the anticipation of a reward or punishment is involved. Additionally, the study explored the effects of stable personality traits and environmental characteristics on risky decision-making. The rRST [9] served as the theoretical framework, offering insights into typical behavior depending on the environment’s characteristics. The VR stimuli were carefully designed to elicit the experience of potential and actual threat or reward, with the key measures focusing on the number of steps taken, time to make the decision, and the average score achieved. Emotional arousal during the VR task was assessed by the three indicators: heart rate, skin conductance, and respiratory rate. As part of the experimental manipulation, negative feedback was introduced at a specific point in the game (10^th^ bifurcation), where participants experienced the falling off the bridge and losing all previously collected points.

The results provide valuable insight into the effects of negative feedback on behavior in the VR environment. Specifically, the significant changes in three out of four VR measurements following the negative feedback suggest a shift toward a more cautious yet more proficient and efficient strategy. This behavioral shift is marked by an increased number of smaller steps, faster movement, and shorter decision times, especially in situations involving double-risky choices, such as bifurcations with two bridges. The shorter decision time after the negative feedback implies that participants adapted to the falling scenario, and their decision-making became more streamlined as they progress in the game. However, the results reveal that observed decrease in overall decision-making time after negative feedback was not simply a consequence of repetitive exercise. This conclusion is supported by the finding that more complex decisions, particularly those involving bifurcations with two bridges required more time from participants. In other words, while participants became more efficient in general, they continued to invest additional time and cognitive resources in situations that demanded more careful consideration of options, such as bifurcations with higher risk.

At the beginning, the participants were more willing to take risks, showing a behavior consistent with the prospect of gaining substantial reward, demonstrating the evaluative trade-off between a potential reward and a risk [1]. However, as the game progresses and participants encounter negative feedback, a noteworthy shift in strategy was observed. Specifically, there was a notable tendency to favor options with larger rewards when faced with risky choices, particularly as the threat of falling became a real and known factor. This strategic choice appears to be linked to a heightened awareness of the stakes involved, indicating a shift in decision-making dynamics after the experience of negative feedback. In general, these results support previous research that revealed approach and avoidance as the two main strategies for coping with mediated threats in the VR environment, with sensation seeking as one of the main factors influencing the employed coping strategies [40]. However, our research has shown that BAS, most similar to the concept of sensation seeking, was not a significant predictor, most probably due to the fact that participants were more focused on actual, rather than potential gain or loss, which is then more reflected through the activation of fight (approach) and freeze (avoidance) behaviors.

Regarding the role of BAS and other personality traits, our research has revealed some potential issues related to research design. We anticipated that the number of points collected would be recognized as a reward, yet this particular VR measure did not correlate with any of the psychological variables. In contrast, BAS was significantly negatively correlated with the time taken to finish the game, especially after the negative feedback. This suggests that participants, particularly those with high BAS scores, may have perceived completing the game as the most relevant reward. This interpretation is further supported by the fact that participants generally played faster and made quicker decisions after the bridge collapse. Previous research suggests that time pressure, in this case the timer constantly visible in the corner of the screen, makes individuals less sensitive to reward values and less likely to choose options with higher uncertainty [14]. BAS appears to be a complex mechanism with multiple facets, not solely reducible to reward responsiveness, and thus requires careful selection of objective measures to accurately capture its role in decision-making processes [41].

The analysis of physiological measures supports the validity of the experimental procedure and confirms the VR environment as a credible simulation for examining these phenomena. After the negative feedback, the results revealed heightened arousal levels, evidenced by deepened breathing and increased skin conductance. Although no significant changes were observed in the heart rate before and after the negative feedback, it is posited that subjects were already highly excited (average heart rate was 93.36) due to the VR experiment itself, potentially reaching a "ceiling" in the heart rate frequency, thereby mitigating significant variations. It is worth mentioning that our results regarding the change in skin conductance level in stressful situations are not in line with previous research. Participants in our study exhibited a significant increase in skin conductance level (p = .012) in response to stress, whereas a similar VR study reported the opposite effect [31]. The authors of that study attributed the decrease in EDA to participants’ freezing reactions. In our study, a similar effect was observed with skin conductance rate amplitude, which correlated negatively with Freeze. Conversely, both respiratory rate and heart rate amplitudes showed positive correlations with Freeze following the negative feedback. This discrepancy raises questions about potential confounding effects of the instruments used, as well as the specific characteristics of the VR environments employed.

The presented results revealed some gender differences in physiological responses and VR behavior. Comparing the periods before and after negative feedback, females exhibited significant differences in skin conductance level and respiratory rate amplitude, whereas males demonstrated significant difference in heart rate span. This result can be partially attributed to females being more provoked by the experimental situation itself, as manifested in a significantly higher heart rate span even before the bridge collapse. Females were generally more excited throughout the whole VR experiment, while males’ heart rates surged after the negative feedback and points loss. Conversely, following the bridge collapse, females experienced a notable increase in breathing amplitude and skin conductivity as physical manifestations of physiological arousal, which was not observed in males. On the other hand, we were not able to confirm previous research indicating that males tend to manifest riskier behavior, particularly under stress [42]. Although females in our experiment were generally more physiologically aroused, they were choosing bridge crossings over the safe paths significantly more often than males, both before and after the bridge collapse.

Our results support some previous findings indicating that the change in respiratory rate is the most evident signal of somatic anxiety [32]. Participants in our study primarily associated respiratory rate amplitude with their subjective assessment of arousal. Breathing depth was the parameter that people are most aware of, in contrast to the more discrete heart rate changes that requires attentive self-monitoring, or skin conductance level, which individuals generally remain unaware of [43]. Additionally, our results have shown that subjective estimate of arousal is significantly correlated with the perceived realism of the VR environment, and negatively with the frequency of playing computer games. Future research using VR environments should consider participants’ expectations regarding the quality of graphics and animation, particularly for those with extensive experience in high-end computer games. This is especially crucial when assessing performance in VR environments involves wayfinding tasks, as it did in our study [44].

It was expected that risky decisions are a complex process, encompassing previously learned patterns of behavior reflected in personality traits, varying risk levels of situations, and situational arousal influencing specific responses. In the context of the VR game, we hypothesized that behavioral patterns in the initial phase would be more influenced by stable personality traits, while in the latter phase, following negative feedback, they would be more contingent on situational characteristics. Our results supported this hypothesis in relation to specific measures of VR behavior. Prior to the negative feedback and loss, the number of steps as a behavioral measure in the VR environment was predicted by Fight and Freeze, along with the respiratory rate amplitude. A greater number of steps was associated with the lower Fight and higher Freeze, indicating that individuals prone to withdrawal reactions in the face of potential danger and risk tended to adopt a more cautious strategy. Individuals displaying a more pronounced Freeze response in situations where risk is unavoidable, along with the potential for loss, tended to react more cautiously, taking a greater number of small steps and "clicks." This behavior was accompanied by heightened tension, as indicated by an increased respiratory rate amplitude. Conversely, low defensive struggle, indicative of a form of aggressiveness, seemed to be avoidant in confronting risky situations. However, after the negative feedback, the characteristics of the situation or the task (fall of the bridge, loss of all points) became more important than basic predispositions for predicting future behavior. Respiratory rate amplitude remained a significant predictor of arousal, while other rRST personality traits were not related to the game strategy selection, at least in the game scenario used in this study.

Personality traits seem to shape how the tendency towards risky behavior will manifest, as suggested by previous studies [6]. However, when the context in which a person finds himself is intense, as in the case of this VR game scenario, situational factors and time pressure along with the potential risk of loss might become more important factors than the stable predispositions. In other words, the impact of the real threats on decision-making can override the influence of the stable personality traits. These findings may have broader generalizability across diverse contexts. For example, in an investigation examining the influence of real threats and personality traits on behavior during cognitive tasks with potential for failure, the results indicated that experimental manipulation such as testing conditions and task difficulty—had a more significant impact than personality traits in predicting behavioral strategies [45]. Furthermore, the assessment of self-efficacy in creating a short questionnaire indicated that personality traits have significant effects only before the received random feedback, including positive, negative, and neutral evaluation. After the feedback, self-efficacy assessment is not related to personality traits, but to the random evaluation of the tasks [46]. These insights could have broader applications, emphasizing the importance of considering both stable traits and situational factors in understanding and predicting behavior in various contexts. They offer valuable insights for experimental and personality psychologists by revealing how risk-taking is influenced by situational, emotional, and personality factors. Additionally, they guide VR designers in creating more ecologically valid environments, highlighting VR’s potential as a tool for psychological research.

### Study limitations and future directions

The key strengths of this study lie in its focused investigation of patterns in risky decision-making within the VR environment, when participants anticipate either a reward or punishment. Nonetheless, certain limitations affect the generalization of the results and highlight important directions for future research. Firstly, our examination of decision-making occurs within the controlled laboratory setting, offering only a partial reflection of real-life decision-making scenarios. Future studies could incorporate more ecologically valid settings to enhance the applicability of findings to real-life decision scenarios. Although the participants, particularly the females, have estimated the environment as sufficiently realistic, additional improvement of the VR game in the sense of higher resolution and more advanced, but also more hardware demanding assets, could further improve participants’ VR presence and immersion.

Secondly, our participants were young and educated volunteers, and we acknowledge that this may limit the generalizability of findings to the broader population because the tendency to engage in risky activities decreases with aging. To address this concern in future research, it is imperative to broaden the sample by including individuals from a more diverse range of age, as well as cultural, educational, and socioeconomic backgrounds.

Despite these considerations, our results have significant implications for understanding the relationship between personality and situations. It is likely that in situations that do not have an explicitly evaluative character, the individual is guided by stable patterns of behavior. Situational characteristics, such as negative feedback, show greater importance for behavioral strategy than stable personality traits.

## Conclusions

The results show that at the beginning of the VR game, individual tendencies towards risky choices were influenced by personality traits, especially those related to withdrawal reactions. However, after the negative feedback, situational factors took precedence over innate predispositions, shaping decision-making strategies. Physiological measures, including the heart rate, skin conductance, and respiratory rate, confirmed the validity of the experimental manipulation and highlighted the impact of the intense context on arousal levels. These findings contribute to our understanding of how individuals navigate and make decisions in VR environments that simulate potential threats and rewards. This study emphasizes the dynamic interaction between the stable personality traits and situational factors, showcasing the complex nature of decision-making processes. The implications extend beyond the specific VR game scenario, suggesting potential generalizability to diverse contexts where individuals face real or simulated risks. However, our study has also underscored the critical importance of selecting appropriate statistical models, predictors, and VR measures to accurately capture the complexities of human behavior in immersive environments.
